# pyGOMoDo: GPCRs modeling and docking with python

**DOI:** 10.1093/bioinformatics/btad294

**Published:** 2023-05-03

**Authors:** Rui P Ribeiro, A Giorgetti

**Affiliations:** Computational Biomedicine - IAS-5/INM-9, Forschungszentrum Jülich, Jülich, Germany; Department of Biotechnology, University of Verona, Verona 34134, Italy; Computational Biomedicine - IAS-5/INM-9, Forschungszentrum Jülich, Jülich, Germany; Department of Biotechnology, University of Verona, Verona 34134, Italy

## Abstract

**Motivation:**

We present pyGOMoDo, a Python library to perform homology modeling and docking, specifically designed for human GPCRs. pyGOMoDo is a python wrap-up of the updated functionalities of GOMoDo web server (https://molsim.sci.univr.it/gomodo). It was developed having in mind its usage through Jupyter notebooks, where users can create their own protocols of modeling and docking of GPCRs. In this article, we focus on the internal structure and general capabilities of pyGOMoDO and on how it can be useful for carrying out structural biology studies of GPCRs.

**Results:**

The source code is freely available at https://github.com/rribeiro-sci/pygomodo under the Apache 2.0 license. Tutorial notebooks containing minimal working examples can be found at https://github.com/rribeiro-sci/pygomodo/tree/main/examples.

## 1 Introduction

**Figure 1.**
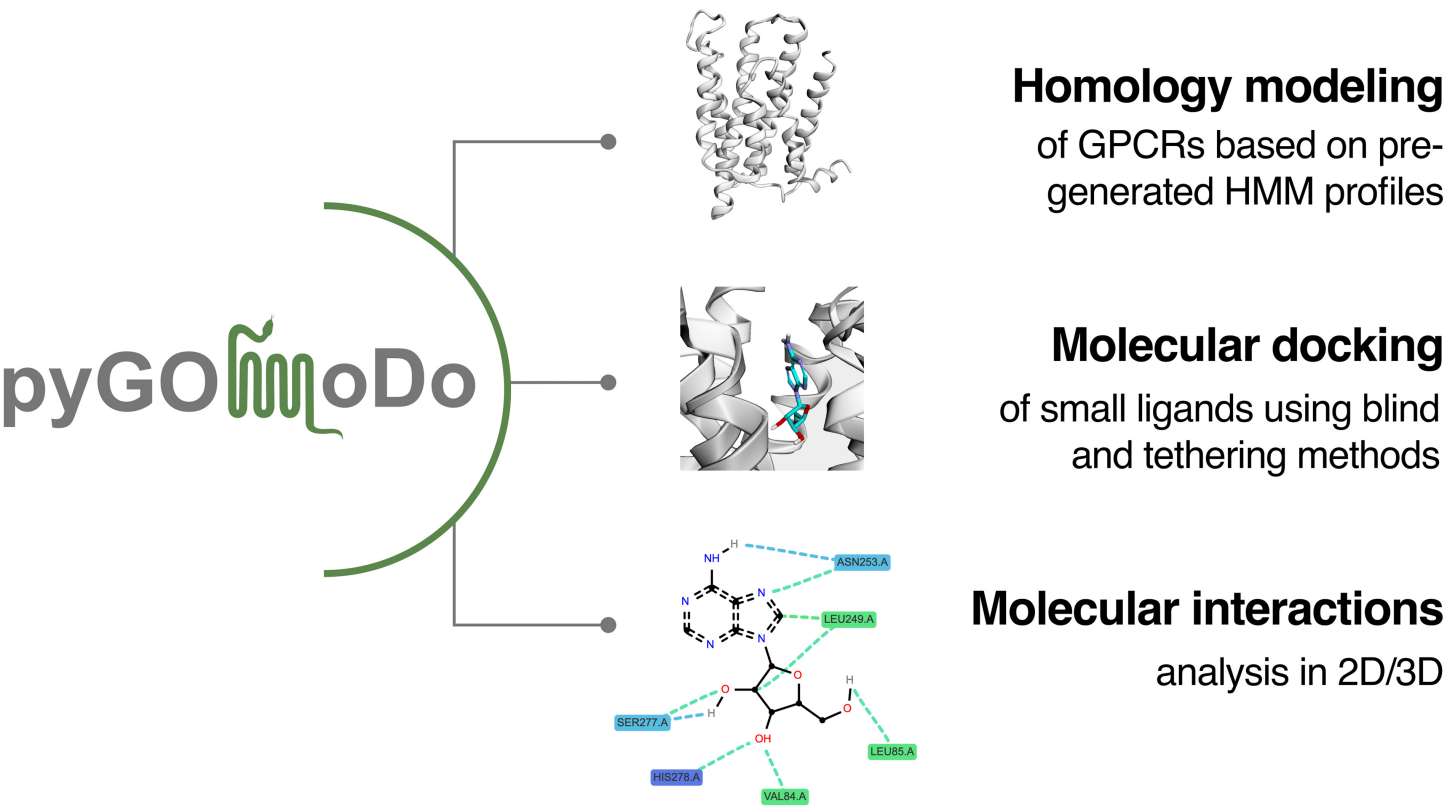
Structure of the pyGOMoDo package.

GPCRs Online Modeling and Docking Webserver (GOMoDo) is a G-protein coupled receptors (GPCRs) online modeling and docking web server, developed by Sandal and collaborators (Sandal et al. 2013), and it is publicly available since 2013 (https://molsim.sci.univr.it/gomodo). With a very easy user interface, this biocomputing platform allows users to effortlessly model GPCR structures and dock ligands to the model, obtaining biological and pharmacological relevant data, in a consistent pipeline: protein sequence alignment, homology modeling and model quality assessment, and docking (Sandal et al. 2013). One of the novelties that GOMoDo brought to the bioinformatics community was the use of a curated local database of pre-aligned GPCR sequences, plus the possibility of directly docking the ligands into the models. Moreover, the user has the possibility of uploading models generated by other tools. However, at the time of its development and deployment only less than 3% of the human GPCR structures were available. Moreover, the code behind both the back and front-end of the web server has not only been difficult to maintain over the years, but no longer satisfies the necessary requisites for the utmost web performance and security. Despite these limitations, GOMoDo is still used today, which prompted us to revamp it by creating pyGOMoDo. Compared to its predecessor, pyGOMoDo boasts several noteworthy improvements, such as the addition of a new model quality assessment tool [QMEANBrane (Studer et al. 2014)], multiple options for docking programs (including AUTODOCK VINA (Trott and Olson 2010) and rDOCK (Ruiz-Carmona et al. 2014)), enhanced utilities for interaction analysis [ProLIF (Bouysset and Fiorucci 2021)], and up-to-date internal databases (Fig. 1).

GPCRs, which are expressed in every organ system, are one of the most crucial pharmacological targets, playing a significant role in regulating almost every aspect of physiology. Although many big pharmaceutical and biotech companies lost their interest and expertise in GPCR targets (Mullard 2018), the truth is that not only around 20% of FDA approved drugs each year target these receptors, but also a new opening up in GPCR-based drug discovery has been stated (Mullard 2018). In fact, GPCRs are still offering emerging therapeutic opportunities: peptide therapeutics and modification of natural ligands (Rask-Andersen et al. 2011; Hauser et al. 2017; Demartis et al. 2018), GPCRs-target antibody therapeutics (Hutchings et al. 2017), allosteric sites (Thal et al. 2018), orphan human GPCRs (Cohen et al. 2017), oligomerization (George et al. 2002; Ferré 2015), and biased (Oprea et al. 2018) and bivalent (Pérez-Benito et al. 2018) ligands for GPCRs.

Even if the number of three-dimensional structures of GPCRs have been rapidly increasing thanks to the constant improvement in techniques for structure determination like high-throughput X-ray crystallography, multidimensional NMR spectroscopy, Cryo-Electron microscopy (cryo-EM), or small angle X-ray scattering (SAXS) (Pandey et al. 2016), the supply–demand gap in GPCR structures is still enormous. This is mainly due to the hydrophobic nature of the transmembrane domains of GPCRs that make their purification and crystallization challenging (Doerr 2010). Thus, computational protein modeling methods are a valid alternative. Recently, neural networks-based modeling techniques have been shone through, especially since the release of AlphaFold (Jumper et al. 2021) and RoseTTAFold (Baek et al. 2021) (an AlphaFold-based neural network) (Lee et al. 2022). However, even if such algorithms have achieved substantial success, GPCRs modeling template-based methods, pyGOMoDo in particular, still remains as a valid alternative to build high quality structural models (Fierro et al. 2017, 2019; Capaldi et al. 2018; Schneider et al. 2018; Meyer et al. 2022).

When working with modeling and docking programs, it is typically necessary to have a thorough understanding of the software's internal workings. To address this challenge, object-oriented libraries that encapsulate essential functionalities may provide a consistent working interface, enabling users to easily create analysis tools without having to spend time and effort re-implementing standard functionalities. Here, we present the pyGOMoDo: a python library to perform homology modeling and molecular docking of GPCRs. We have built the library from scratch having in mind the key features of the original GOMoDo. Although pyGOMoDo is not a web server, we have chosen to keep its original name to facilitate recognition and establish a connection with its earlier versions.

## 2 Software implementation

The library is divided into two main modules: the modeling and docking modules. The modeling module follows the same framework pipeline as the original web server: protein sequence alignment and hidden Markov model profile generation with the hh-suite (Steinegger et al. 2019), and homology modeling with the Modeller software (Webb and Sali 2016). We have included in pyGOMoDo a new model quality assessment. The models can now be assessed with the QMEANBrane program (Studer et al. 2014) through the Swiss-Model web server API (Waterhouse et al. 2018). The HMM profiles are generated against the most updated version of the UniRef30 (Mirdita et al. 2017) database, and the templates are obtained by aligning the generated HMM profile against a database of HMM profiles for the GPCRs whose structures are known. We constructed the latter database from the ground up by gathering all human GPCR structures available on the Protein Data Bank (rcsb.org) (Berman et al. 2000). All the structures were parsed, cleaned, and uniformed, and their HMM profiles were built also against the UniRef30 database (Mirdita et al. 2017). We have computed the coverage (the percentage of solved amino acids in the sequence) for each PDB structure and, whenever feasible, obtained information about the receptor's conformational state. Thus, the choice for the best templates for modeling can be done according to the sequence identity shared with the template and considering the “solved coverage” of the structure and/or its conformational state (when possible).

Differently from the original GOMoDo web server, the docking module of pyGOMoDo works independently from the modeling module. The user can, therefore, perform molecular docking over a structure modeled with the modeling module, another software or web server, or even over an experimental structure.

The docking module implements the Autodock Vina (Trott and Olson 2010) and rDOCK (Ruiz-Carmona et al. 2014) docking programs in three different docking protocols: (i) a typical Autodock Vina protocol, but with automatic selection of the orthosteric binding site (all the Autodock Vina setting values can be toggled by the user), (ii) docking of molecules on the binding site of crystallographic ligand with rDock, and (iii) tethered docking of molecules on a crystallographic ligand also with rDOCK. The docking module also implements the Protein–Ligand Interaction Fingerprints (ProLIF) (Bouysset and Fiorucci 2021) tool that allows the analysis of all the ligand-protein interactions.

The pyGOMoDo library was specifically designed having in mind its usage through Jupyter notebooks providing the sweet-spot between performance, flexibility, and visualization [tables visualization are rendered by the pandas (Mckinney 2010) library and protein visualization by the py3Dmol (Rego and Koes 2015) library]. The pyGOMoDo code and its documentation and tutorials can be accessed through https://github.com/rribeiro-sci/pygomodo/tree/main/examples. However, since all the software dependencies are already precompiled inside a docker container, we recommend using pyGOMoDo through the latter, available at https://hub.docker.com/r/rpribeiro/pygomodo.

## 3 Conclusion

Taken as a whole, here we present an updated tool, pyGOMoDo, to perform homology modeling and docking on GPCRs. By developing a python library instead of a static web server, we are offering a much more flexible tool, so users can create their own modeling and docking pipelines according to their needs. The user has the flexibility to intervene at various stages of the process. For example, for the docking component, the software allows the utilization of models or structures generated via alternative methods, such as AlphaFold2 (Jumper et al. 2021) and RoseTTAFold (Baek et al. 2021). Indeed, as for homology models, AlphaFold2 now allows the prediction of different conformational sites of GPCRs (del Alamo et al. 2022; Stein and Mchaourab 2022; Pándy-Szekeres et al. 2023). Thus, models created by independent modeling programs/algorithms can be uploaded and funneled to the different docking protocols offered by pyGOMoDo.
